# Assessing cellular energy dysfunction in CFS/ME using a commercially available laboratory test

**DOI:** 10.1038/s41598-019-47966-z

**Published:** 2019-08-07

**Authors:** Cara Tomas, Tiffany A. Lodge, Michelle Potter, Joanna L. Elson, Julia L. Newton, Karl J. Morten

**Affiliations:** 1 Institute of Cellular Medicine, Newcastle University, Newcastle upon Tyne, UK; 2 Nuffield Department of Women’s and Reproductive Health, University of Oxford, John Radcliffe Hospital, Oxford, UK; 3 Institute of Genetic Medicine, Newcastle University, Newcastle upon Tyne, UK; 4 Centre for Human Metabolomics, North-West University, Potchefstroom, South Africa; 5 Newcastle upon Tyne Hospitals, NHS Foundation Trust, Newcastle upon Tyne, UK

**Keywords:** Metabolomics, Analytical biochemistry

## Abstract

The mitochondrial energy score (MES) protocol, developed by the Myhill group, is marketed as a diagnostic test for chronic fatigue syndrome/Myalgic Encephalomyelitis (CFS/ME). This study assessed the reliability and reproducibility of the test, currently provided by private clinics, to assess its potential to be developed as an NHS accredited laboratory test. We replicated the MES protocol using neutrophils and peripheral blood mononuclear cells (PBMCs) from CFS/ME patients (10) and healthy controls (13). The protocol was then repeated in PBMCs and neutrophils from healthy controls to investigate the effect of delayed sample processing time used by the Myhill group. Experiments using the established protocol showed no differences between CFS/ME patients and healthy controls in any of the components of the MES (p ≥ 0.059). Delaying blood sample processing by 24 hours (well within the 72 hour time frame quoted by the Myhill group) significantly altered many of the parameters used to calculate the MES in both neutrophils and PBMCs. The MES test does not have the reliability and reproducibility required of a diagnostic test and therefore should not currently be offered as a diagnostic test for CFS/ME. The differences observed by the Myhill group may be down to differences in sample processing time between cohorts.

## Introduction

Chronic Fatigue Syndrome/Myalgic Encephalomyelitis (CFS/ME) affects 250,000 people in the UK^1^. With an unknown cause and currently no accredited diagnostic tests or widely effective treatment options, focusing on physiological symptoms treatment management remains difficult^1^. The uncertainty around the aetiopathogenesis of the disease and lack of known biomarkers means there are currently no accredited diagnostic tests. This is problematic not only for diagnosis but when assessing new treatments in clinical trials^2^. Studies suggest that CFS/ME may be associated with abnormalities of energy production or mitochondrial function^3–6^. The Mitochondrial Energy Score procedure was developed by Myhill *et al*. and first published in 2009^7^. Subsequently it has been described as a test which should be made available to all CFS/ME patients and used as part of treatment management^8,9^. The test is currently offered by several private clinics who offer treatment based on the results. A published audit of results confirming the benefits of treatments or that an improvement energy score correlates with patient improvement has yet to be documented. Although not offered by the NHS in the UK, many patients pay for the test privately and approach their NHS clinician with the results. The Myhill test aims to correlate neutrophil mitochondrial dysfunction, measured as total cellular ATP, to the severity of disease in CFS/ME patients^7–9^. If the Myhill test can be confirmed as a specific and reliable test for mitochondrial dysfunction in CFS/ME it would be a great benefit to patients in management of their symptoms and assessing the effectiveness of new clinical treatments.

In order to add robustness to our approach, teams in two separate laboratories (Newcastle & Oxford) were invited to set up the Myhill protocol independently. The aims of this study were two-fold. Firstly, we set out to follow the Myhill protocol in order to determine if live cells isolated from whole blood can be reliably used to assess mitochondrial function and whether this approach has the potential to be developed as an NHS accredited laboratory test. Then we went on to explore whether the conditions recommended for transport and storage, and the cell type used, could have influenced test outcome.

## Results

### Participant characteristics

Table 1 shows the number of participants, age and gender for the control and CFS/ME groups in this study.

**Table 1:** Participant characteristics.

	Number of participants	Age (mean ± SD)	Gender (M:F)
Healthy control	13	39.1 ± 12.6	4:9
CFS/ME	10	38.7 ± 15.8	2:8

### Myhill test

**Figure 1:**
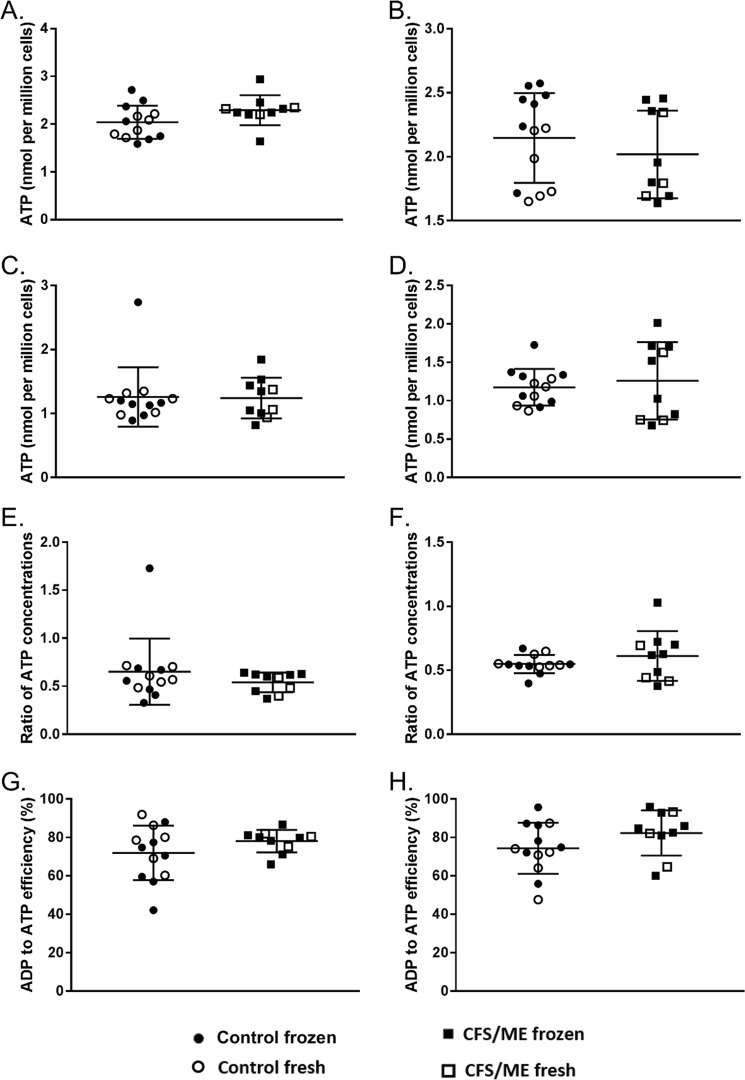
ATP parameters calculated using the protocol from the Myhill test. ATP concentration in (**A**) neutrophils (p = 0.295) and (**B**) PBMCs (p = 0.059) of CFS/ME patients and healthy controls in the presence of excess magnesium. ATP concentration in the absence of excess magnesium in (**C**) neutrophils (p = 0.737) and (**D**) PBMCs (p = 0.947). Ratio of ATP concentration in cells with endogenous magnesium to ATP concentration in cells with excess magnesium in (**E**) neutrophils (p = 0.337) and (**F**) PBMCs (p = 0.314). ADP to ATP efficiency in (**G**) neutrophils (p = 0.054) and (**H**) PBMCs (p = 0.550). CFS/ME n = 10; control n = 13. Groups were compared using Welch’s t-tests.

For each of the parameters calculated in this study using the MES protocol results are shown as a combination of fresh and frozen cells for the healthy control and CFS/ME cohorts. There were no significant differences between fresh and frozen neutrophils or PBMCs for any of the parameters in either healthy controls or CFS/MS patients (Supplementary Information). Therefore, the fresh and frozen samples were pooled for the experiments described below and shown in Fig. 1.

#### ATP concentration in the presence of excess magnesium

The first experiment investigated the ATP concentration in neutrophils and PBMCs in the presence of excess magnesium in CFS/ME patients and healthy controls (Fig. 1A,B). Magnesium is required for the intracellular production of ATP. The addition of magnesium to the cells eliminates the effect of differing inter-participant magnesium levels. Results showed there to be no difference in the concentration of ATP between CFS/ME patients and controls in either neutrophils (p = 0.295) or PBMCs (p = 0.059).

#### ATP concentration in the presence of endogenous magnesium

ATP concentration was investigated in the absence of excess magnesium. Magnesium deficiencies have previously been reported in a subset of CFS/ME patients^10,11^, however, this was not shown in all studies investigating magnesium status in CFS/ME^12^. Magnesium is required as a co-factor for the production of ATP, therefore, the measurement of ATP production with only endogenous magnesium present in the cells gives a more accurate indication of the ATP actually being produced by the cells as opposed to the potential capacity the cells have for ATP production which is shown when excess magnesium is added^13^. Results showed there to be no difference in the concentration of ATP between CFS/ME patients and controls in either neutrophils (p = 0.737) or PBMCs (p = 0.947) (Fig. 1C,D).

#### ATP ratio

A ratio was calculated of ATP with endogenous magnesium to ATP with excess magnesium. This shows the proportion of ATP that is available as an energy supply as it is the proportion of ATP complexed with magnesium to form MgATP. The ratio was calculated for CFS/ME patients and controls in both neutrophils and PBMCs. There was no significant differences in either cell type (p = 0.337 and p = 0.314 respectively), Fig. 1E,F.

#### ATP to ADP efficiency

**Figure 2:**
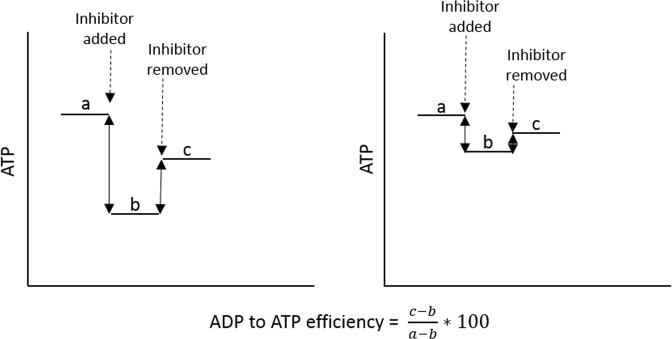
Graphs of equal ADP to ATP efficiency with different profiles. This shows the importance of looking at the % drop in ATP after the addition of the inhibitor sodium azide to look at the reliance of cells on OXPHOS.

The final part of the Acumen protocol studied involved investigating the efficiency of the conversion of ADP to ATP. This gives us an estimate of the efficiency at which ADP can be recycled to form ATP to provide cellular energy. There were no significant differences between the CFS/ME and control cohorts in either the neutrophil (p = 0.054) or PBMCs ADP to ATP efficiency (p = 0.550), Fig. 1G,H. In their protocol Acumen refer to this parameter as ‘ADP to ATP efficiency’ however, in their publications on this work^7–9^ they refer to the same parameter as OXPHOS. The label OXPHOS is misleading as the equation relies on the recovery rate after the inhibitor has been removed and therefore while OXPHOS is a component of the equation, it is not represented by the final numerical value. As long as the % recovery rate is the same between two samples then they will have the same value of ADP to ATP efficiency regardless of the % decrease in ATP caused by the addition of the OXPHOS inhibitor as illustrated in Fig. 2. This parameter cannot be called OXPHOS, as it is in the papers published by the Myhill group.

**Figure 3:**
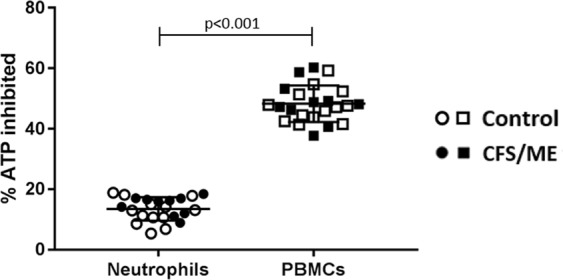
Percentage of ATP inhibited in neutrophils and PBMCs when OXPHOS inhibitor sodium azide was added. Calculated as the amount of ATP after the addition of sodium azide as a percentage of ATP before cells are treated. This shows how reliant the cells are on OXPHOS as an energy source. A lower % ATP inhibition shows a lower reliance on OXPHOS. N = 23. There were no significant differences between CFS/ME and healthy control % ATP inhibition for either cell type (Supplementary Information S3).

The % ATP inhibited (the first component of the ADP to ATP efficiency equation) may be used as a more accurate representation of OXPHOS as it shows the % of ATP inhibited when OXPHOS is inhibited and therefore gives an indication of OXPHOS. However, the value of ATP made by mitochondria after the inhibitor is removed (c) will be influenced by substrate flow into the mitochondria. Hence a reduction in (c) may not be a direct effect of mitochondrial dysfunction but could be linked to substrate supply into the mitochondria. % ATP inhibited is used in Booth *et al*. and Myhill *et al*.^9^, although it is still used alongside the “OXPHOS” parameter in these publications^8,9^. The % ATP inhibited can often tell a vastly different story than ADP to ATP efficiency (called OXPHOS by Myhill *et al*.) with neutrophils and PBMCs showing similar values for ADP to ATP efficiency (Fig. 1G,H) but very different values for % ATP inhibited by sodium azide (Fig. 3) indicating differing pathways of ATP production in the two cell types. Figure 3 shows that a significantly higher % of ATP is inhibited in the PBMCs than in neutrophils which suggests that PBMCs in freshly isolated blood rely on OXPHOS for ATP production to a much greater extent that neutrophils (p < 0.001). This is in keeping with other previous work^14–18^ that has shown neutrophils to be predominantly glycolytic cells.

#### ADP/ATP translocator protein

The part of the protocol investigating ADP/ATP translocator activity was not able to be conducted. Key reagents specified in the Acumen protocol for these experiments were no longer available. Replacements were offered for these products on the manufacturers website, however, as the purpose of this study was to investigate the validity of the Acumen protocol in the diagnosis of CFS/ME, it was decided that it would be inappropriate to use other reagents not specified by the Acumen protocol.

### Effect of delayed cell isolation

**Figure 4:**
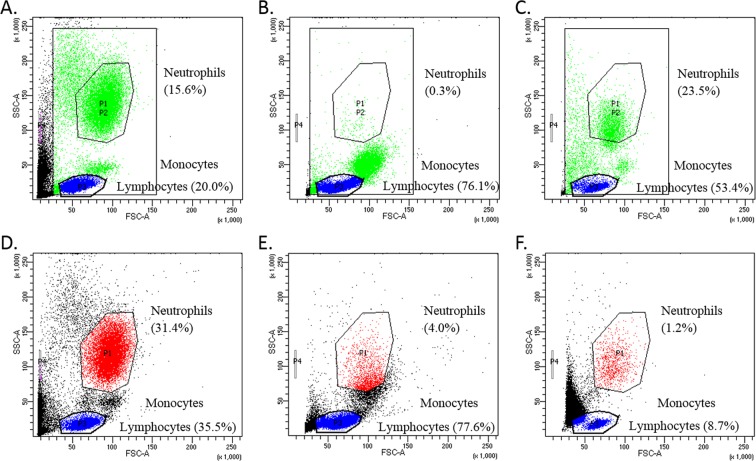
Cells were analysed using FACS. (**A**) shows whole blood 1 hr after isolation following RBC lysis. Three distinct populations of cells consisting of neutrophils, monocytes and lymphocytes are observed. (**B**,**C**) were isolated using Histopaque^TM^ (**B**) =PBMC (monocytes plus lymphocytes) and **(C)** =neutrophil fractions 1 hr after isolation. (**D**–**F**) are comparable to (**A**–**C**) but 24 hrs after isolation.

Acumen analyse blood samples that are 24–72 hr post phlebotomy^7^. We had concerns that this time frame was too long for neutrophils which would likely become activated^19,20^ and most of the granulocytes would be lost in that time period which may account for the differences between our results (Fig. 1) and the results shown by Myhill *et al*.^7–9^. CFS/ME patients and controls could also differ in the degree of neutrophil activation over time and as this is an unknown variable could compromise the interpretation of the results (i.e. assessing changes in the collection tube rather than differences between the CFS/ME patients and controls *in vivo*). We isolated the cell fractions from fresh blood and blood that had been left for 24 hrs (similar to Myhill test samples) in order to determine the effect of delayed cell isolation. We first analysed the samples by Flow Cytometry (FACS), a useful tool that separates cells based on their size and internal composition (granularity). The results are shown in Fig. 4.

Figure 4B is the 1 hour PBMC fraction from a Histopaque™ separation and the scatter plot shows no neutrophil like cells. Figure 4C is the 1 hour neutrophil layer from a Histopaque™ separation and it shows contamination with lymphocytes and other cellular debris. Figure 4D–F depict cells that were isolated from blood that had spent 24 hrs in heparin at room temperature. The whole white cell fractions in (A) < 1 hr isolation and (D) 24 hr isolation show different proportions of neutrophils and lymphocytes in the respective quadrants suggesting that the longer processing time has altered cell properties. This could change the properties of the cells in the samples isolated at 24 hrs so they no longer reflect cellular function at the time of harvesting. This further complicates the interpretation of the data as the assumption is made that both controls and ME/CFS patients cells behave in the same way after incubation in a blood tube for 24 hrs. In addition, in the Histopaque™ gradients the 24 hr cell populations neutrophil fraction (F) looks quite different to the 1 hr samples (C) with a large drop in neutrophil numbers. Overall, Fig. 4 highlights the importance of isolating the cells from fresh blood. If blood has been left for an extended period of time cells are lost with further processing using Histopaque™, with additional changes in cell morphology possibly due to activation.

**Figure 5:**
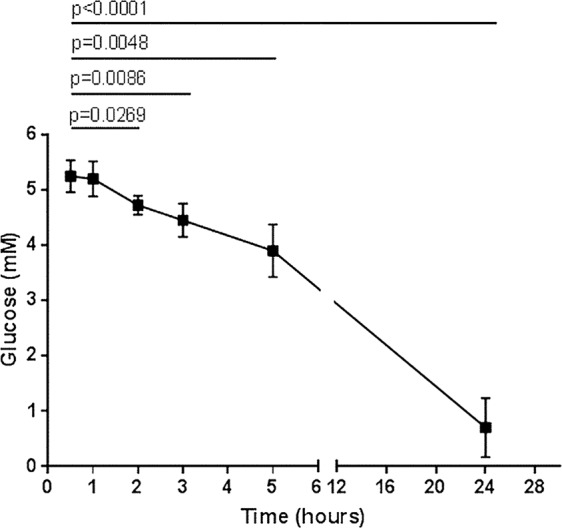
Glucose concentration of whole blood from healthy controls over 24 hours. First reading was within 15 minutes of donation. Control n = 4, groups where compared using Welch’s t-test.

Metabolites linked to the TCA and glycolytic cycles are changed significantly with a general increase in mitochondrial respiration as glucose levels are reduced^21^ and significant changes in metabolism (Morten & Mccullagh Metabolomics unpublished). Once a blood sample is taken as nutrient levels are not replenished and the millions of cells in the sample will deplete nutrients such as glucose over time, which will likely have a significant effect on cellular metabolism. We have studied glucose depletion in control blood samples. In Fig. 5 we demonstrated that in all blood collection systems glucose levels dropped to below 1 mM over 24 hrs. Even after 2 hrs collection blood glucose levels showed a significant drop in glucose levels. It is possible that differences between patients and controls reflect differences generated during prolonged storage and do not reflect differences at the time of sampling.

The results shown in Figs 4 and 5 indicated that these types of experiments should be carried out on cells that had been isolated from fresh blood i.e. <1 hr post sampling, which was not the case for samples used by Myhill *et al*.^7^. It should be noted that all of the samples shown in Fig. 1 were isolated immediately after blood collection.

### Effect of delayed cell isolation on Myhill test

**Figure 6:**
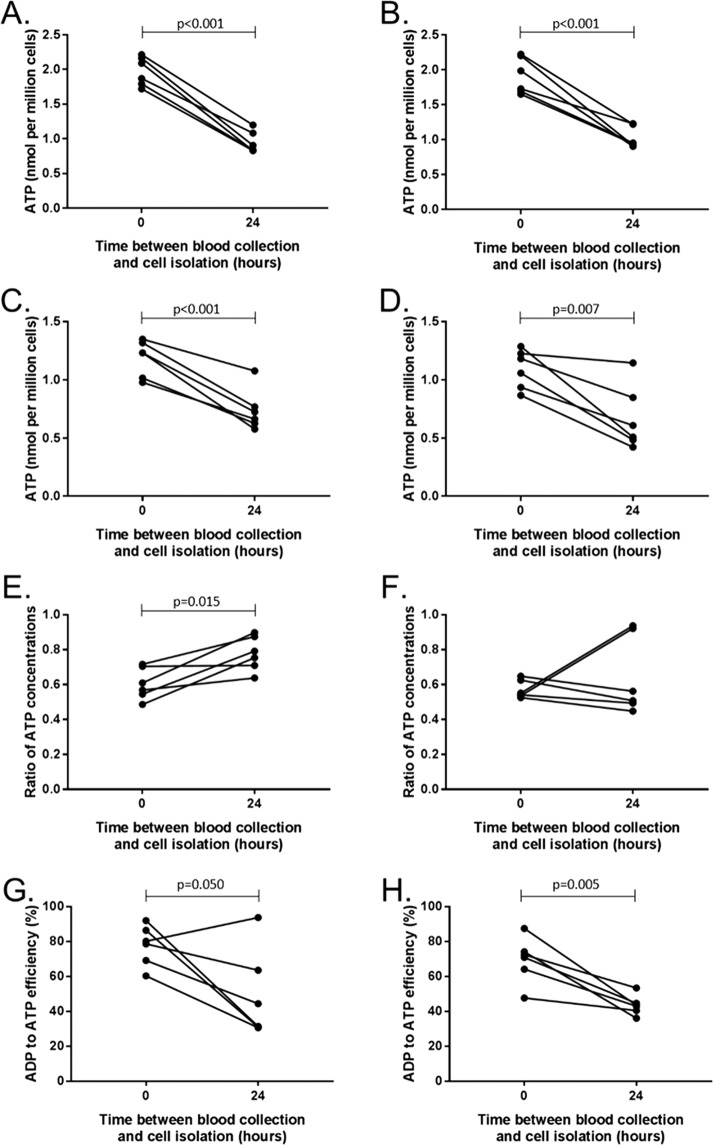
ATP parameters in cells isolated immediately and 24 hours after blood collection. ATP concentration in (**A**) neutrophils and (**B**) PBMCs of healthy controls in the presence of excess magnesium. ATP concentration in the absence of excess magnesium in (**C**) neutrophils and (**D**) PBMCs. Ratio of ATP concentration in cells with endogenous magnesium to ATP concentration in cells with excess magnesium in (**E**) neutrophils and (**F**) PBMCs. ADP to ATP efficiency in (**G**) neutrophils and (**H**) PBMCs. Control n = 6. Groups were compared using paired t-tests.

Given the differences seen between the properties of cells isolated from whole blood at different time points, we repeated the Myhill test using bloods taken from 6 healthy controls with one tube of blood processed immediately and the other tube processed 24 hours after blood collection. The results for each of the parameters for PBMCs and neutrophils are shown in Fig. 6.

The results show a significant reduction in ATP when the cells were isolated 24 hours after collection in neutrophils (p < 0.001) and PBMCs (p < 0.008) in both the excess and endogenous magnesium experiments. Leaving the blood whole for 24 hours also caused a reduction in the ATP ratio in neutrophils (p = 0.015) but not in PBMCs (p = 0.490). The longer sample processing time significantly lowered ADP to ATP efficiency in both neutrophils (p = 0.050) and PBMCs (p = 0.005). This implies that the lower ATP seen in CFS/ME cells by the Myhill group, may be due to the differences in sample handling between the CFS/ME and control cohorts or a difference in controls and CFS/ME cell function over 24–48 hrs. Our results suggest that the fact that the samples from CFS/ME patients are sent through the post and processed between 24–72 hours after blood is taken, while the control blood are collected in the laboratory on the same day as processing, may be the cause of the differences seen between the two cohorts by the Myhill group. An alternative explanation would be the CFS/ME patient and control samples behave very differently during prolonged storage.

Neutrophils, as used by Myhill *et al*., are short lived cells that only last for between 5–90 hours in the circulation^22^. In the context of using neutrophils as an indicator of mitochondrial function it is important to consider that neutrophils in freshly isolated blood sample will be in a non-activated state, which have very different energetic demands to those that have been activated by pathogens^23^.

## Discussion

Given the evidence presented here, we advise that the MES test should not be used as a diagnostic test in its current form as in this study shows there to be no differences between CFS/ME and control results when the MES protocol was followed using fresh blood samples. This is contrary to results from the group who devised the test and offer it to patients.

We explored the impact of delayed sample processing on blood glucose concentration in the collection tube as a possible explanation for the discrepancies in results between our group and the Myhill group. As expected with such high cell numbers the glucose rapidly dropped as the cells utilized the glucose. In addition the neutrophil component on FACS analysis in the white cell fraction showed differences in size and granularity between the 1 hr and 24 hr fractions suggestive of altered properties. Having taken into account a 24 hour delay between blood collection and cell isolation, we have shown decreases in ATP parameters in control cells similar to those seen by the Myhill group in the CFS/ME patients. We suggest that it is potentially the delay between sample collection and cell isolation that is causing the decrease in mitochondrial function previously reported in CFS/ME patients.

While this study used relatively small samples sizes compared with the original study, abnormalities in CFS/ME patients should be reproducible even in small sample sizes given the current use of this test for diagnostic purposes.

The Myhill group have recently altered their protocol to use PBMCs instead of neutrophils, however, this research has not been published and we have no information on the control ranges used and whether they were developed from blood samples processed over 24 hrs. The CFS/ME PBMC study by Tomas *et al*. did show the utility of using PBMC using the Seahorse extracellular flux analyser to study energetics^3^. However, the sudden switch to using PBMCs for the MES protocol appears to be as a result of criticism over the use of neutrophils rather than being an evidence lead change. There has been no data published from the Myhill group regarding the suitability of PBMCs with their previously established protocol, or any publication of results with the new cell type. After a diagnosis of CFS/ME is made using the MES test, patients are subsequently sold supplements in order to treat their CFS/ME, despite there being no placebo-controlled trial to show their effectiveness. The first peer-reviewed publication regarding the MES test from the Myhill group came after they had already been using the test and supplement regime with CFS/ME patients despite there being no published evidenced of the effectiveness, reliability, or reproducibility of the test. Additionally, the MES test has not been conducted using other patient groups with fatigue as a core symptom, therefore its specificity for CFS/ME has not been confirmed.

If energetic dysfunction is to be used as a marker of CFS/ME its exact role in the condition needs to be better understood including studying energetic dysfunction in other fatigue groups. Only when we have clearer understanding of the disease process and knowledge of specific factors shown to be different in CFS/ME should we consider developing a diagnostic test to aid in treatment strategies and determining outcome in clinical trials.

Clinicians approached by patients with results from the MES test should be advised to interpret the results with caution, while patients considering paying for the test should be advised of the lack of supporting scientific evidence. The test in its current form does not have the reliability or reproducibility required of a diagnostic test and therefore should not be offered by the NHS or private clinics as a diagnostic test for CFS/ME. Other tests of energetic dysfunction could be developed using the seahorse extracellular flux assay but more research is required as to the meaning of the results in the aetiology of CFS/ME before a test using this approach should be developed.

## Methods

A 2016 version of the Myhill protocol was obtained from John McLaren-Howard at Acumen Ltd and followed with two exceptions: PBMCs were also collected alongside the neutrophil fraction from the Histopaque™ density gradient and both fresh and cryopreserved cells were used. The use of fresh and cryopreserved samples was born out of necessity as samples could not be collected in a short enough time frame to conduct the experiments at the same time for all CFS/ME samples and controls. Two reagents (Sigma M3260 and P7682) required for the Mitochondrial ATP/ADP Translocator assay are longer available therefore this assay was not carried out.

### Ethical approval

Blood samples were obtained from patients fulfilling the Canadian consensus diagnostic criteria for CFS/ME and healthy controls after obtaining ethical approval from the National Research Ethics Committee North East – Newcastle & North Tyneside (12/NE/0146) (ME/CFS) and County Durham & Tees Valley (12/NE/0121) (controls)^24^. All methods were performed in accordance with their relevant guidelines and regulations. Blood samples were obtained after obtaining informed written consent from all participants.

### Cell Isolation – histopaque™ density gradient

All reagents and equipment were used at room temperature. Blood was collected in lithium heparin tubes. Cells were either isolated from whole blood immediately after sample collection, or 24 hours later as indicated in the results section. The samples in the Myhill studies had been collected at least 24 hrs (up to 72 hours) prior to processing and sent for analysis via the postal service^7^. Neutrophils and PBMCs were separated using a Histopaque™ density gradient.

### The Myhill protocol

#### ATP assays with and without magnesium

ATP concentrations were determined using the ATP Bioluminescent Assay Kit (FLAA) (contains magnesium from Sigma Aldrich (Dorset, UK). Each test was run in quadruplicate. For experiments looking at ATP concentration in the presence of endogenous magnesium the ATP assay mix FL-MM (Sigma Aldrich) was used to prepare a magnesium free solution the assay repeated as before.

#### ADP to ATP efficiency

The efficiency of the conversion of ADP to ATP was monitored in the presence of excess magnesium with and without the complex IV inhibitor sodium azide. ATP concentration was determined as described previously. Following treatment with the inhibitor the cell suspension solution was centrifuged to remove the inhibitor and the pellet resuspended in SBS (combination of NaCl and KCl phosphate buffered to pH 7.8). This solution was left at room temperature for 3 minutes before being centrifuged again and the supernatant removed. The pellet was resuspended in SBS and ATP concentration determined as described previously giving the concentration of ATP generated when the inhibitor had been removed. The aim being to compare the amount of ATP generated from cells in the presence of a respiratory chain inhibitor and when it is washed out.

#### Calculations

A ratio of ATP concentration with endogenous magnesium to ATP concentration in the presence of excess magnesium was calculated using the following equation:

$${\rm{Ratio}}=\frac{{\rm{ATP}}\,{\rm{concentration}}\,{\rm{with}}\,{\rm{endogenous}}\,{\rm{magnesium}}}{{\rm{ATP}}\,{\rm{concentration}}\,{\rm{in}}\,{\rm{the}}\,{\rm{presence}}\,{\rm{of}}\,{\rm{excess}}\,{\rm{magnesium}}}$$

The equation used to calculate ADP to ATP efficiency was:

$${\rm{ADP}}\,{\rm{to}}\,{\rm{ATP}}\,{\rm{efficiency}}=(\frac{(c-b)}{(a-b)})\times 100$$

Where:

a = Initial ATP concentration as determined in ATP concentration experiment

b = ATP concentration in the presence of the ATP inhibitor

c = ATP concentration after removal of the ATP inhibitor

### Flow cytometry

Cells were prepared for flow cytometry in one of two ways. The first was using a Histopaque gradient to isolate a PBMC fraction and a neutrophil fraction as described earlier and the second method used a red blood cell (RBC) lysing solution (Biolegend 420301) using the manufacturers protocol to give the white cell fraction. The white cell pellet was re-suspended in 500 µL PBS along with the PBMC and neutrophils from the Histopaque isolation, on the BD LSR II flow cytometer. Voltages for the forward scatter (FSC) and side scatter (SSC) were adjusted until distinct population of cells were visible on the plot. A low flow rate was used to analyse the samples over a 4 minute time period. This procedure was conducted on blood samples prepared at 1 and 24 hours post collection.

### Glucose monitoring of whole blood

Blood was collected from healthy donors in sodium heparin tubes. Glucose levels were monitored using Accu-Chek Performa Nano (Roche). First reading was within 15 minutes of donation. Vaccutainers where kept at room temperature for the duration of the experiment and periodically gently inverted.

### Statistics

Assays were performed according to the 2016 Acumen protocol in both neutrophils, as stated in the protocol, and PBMCs obtained from 10 CFS/ME patients and 13 control subjects. Statistics were calculated using un-paired Welch’s t-tests after testing for normality. For comparisons between cells isolated immediately after blood collection and 24 hours later, paired t-tests were used.

## Supplementary information

Supplementary information

## Data Availability

The datasets used in this study are available from the corresponding author on reasonable request.
