# Supplementary material for: Assessing cellular energy dysfunction in CFS/ME using a commercially available laboratory test
Source: Sci Rep. 2019 Aug 7;9:11464. doi: 10.1038/s41598-019-47966-z (PMC6686017; doi:10.1038/s41598-019-47966-z)
Supplement: Supplementary file 1 — Supplementary information [file 41598_2019_47966_MOESM1_ESM.pdf]

Assessing cellular energy dysfunction in CFS/ME using a commercially available laboratory test

Cara Tomas, Tiffany A Lodge, Michelle Potter, Joanna L Elson, Julia L Newton, Karl J Morten

Supplementary Information

The impact of cryogenic storage on the level of ATP determined in neutrophils and PBMCs was assessed. It was determined that there was no significant difference between the stored and immediately processed neutrophils or PBMCs with regards to their ATP content in healthy control or CFS/ME cells (Fig S1 & S2). As such, the fresh and frozen samples were pooled for each cohort for the subsequent experiments shown in the main manuscript (fig 1).

Effect of freezing on healthy control PBMCs and neutrophils

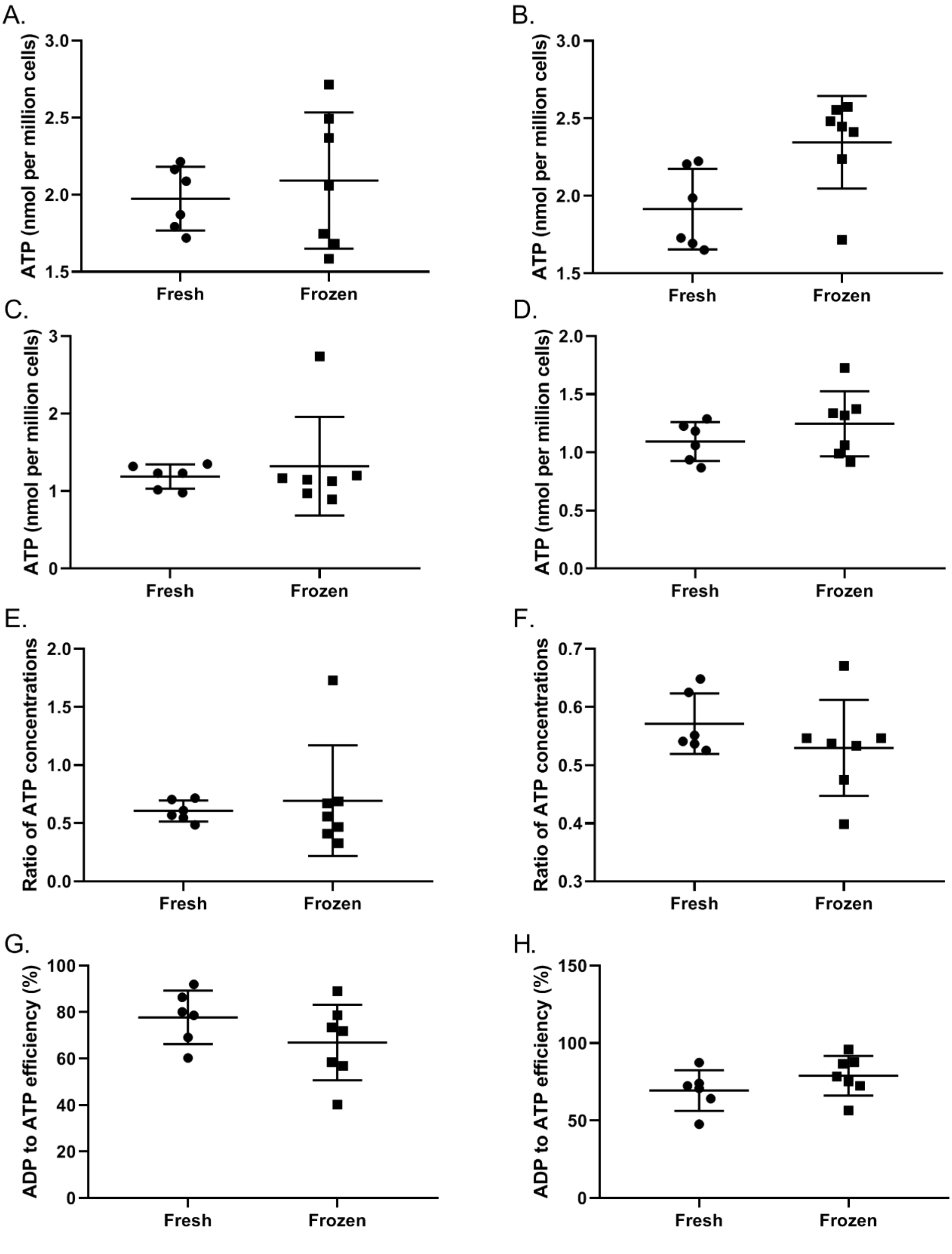

***Figure S1. Results from an ATP profile test conducted using fresh (n=6) and frozen (n=7) healthy control neutrophils and PBMCs**. ATP concentration in **(A)** neutrophils (p=0.546) and **(B)** PBMCs (p=0.019) of healthy controls in the presence of excess magnesium. ATP concentration in the absence of excess magnesium in **(C)** neutrophils (p=0.629) and **(D)** PBMCs (p=0.268). Ratio of ATP concentration in cells with endogenous magnesium to ATP concentration in cells with excess magnesium in **(E)** neutrophils (p=0.667) and **(F)** PBMCs (p=0.309). ADP to ATP efficiency in **(G)** neutrophils (p=0.201) and **(H)** PBMCs (p=0.211). Groups were compared using Student's t-tests. Significance level p≤0.01.*

Effect of freezing on CFS/ME PBMCs and neutrophils

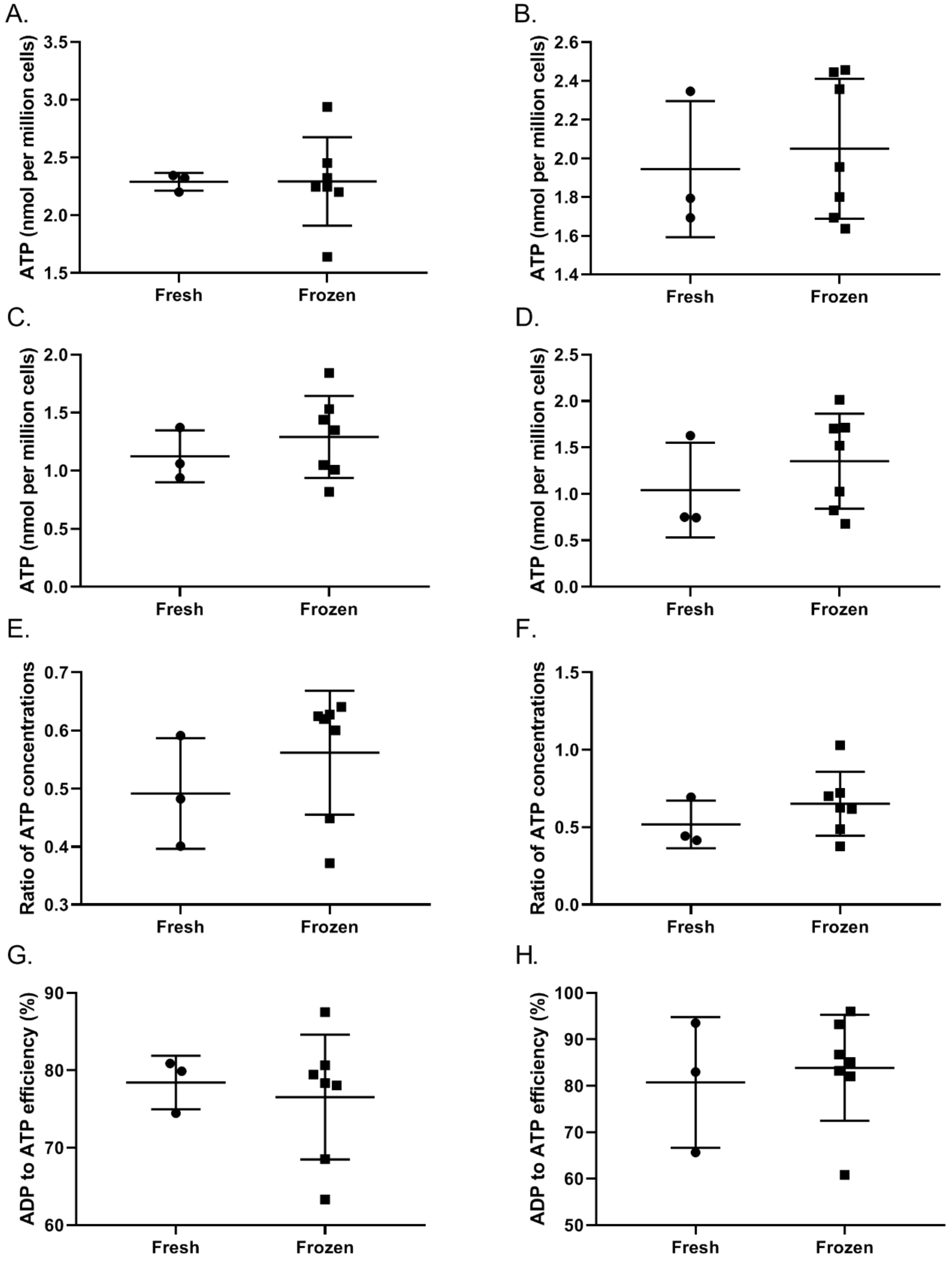

Figure S2. Results from an ATP profile test conducted using fresh (n=3) and frozen (n=7) CFS/ME neutrophils and PBMCs*. ATP concentration in* ***(A)*** *neutrophils (p=0.990) and* ***(B)*** *PBMCs (p=0.683) of CFS/ME patients in the presence of excess magnesium. ATP concentration in the absence of excess magnesium in* ***(C)*** *neutrophils (p=0.481) and* ***(D)*** *PBMCs (p=0.401). Ratio of ATP concentration in cells with endogenous magnesium to ATP concentration in cells with excess magnesium in* ***(E)*** *neutrophils (p=0.356) and* ***(F)*** *PBMCs (p=0.348). ADP to ATP efficiency in* ***(G)*** *neutrophils (p=0.716) and* ***(H)*** *PBMCs (p=0.715). Groups were compared using Student's t-tests. Significance level p≤0.01.*

Comparing the percentage of ATP inhibited in neutrophils and PBMCs when OXPHOS inhibitor sodium azide was added between healthy control and CFS/ME patients

There were no differences between CFS/ME and healthy control cohorts for the percentage of ATP inhibited by sodium azide in either neutrophils (p=0.290) or PBMCS (p=0.343). The CFS/ME and healthy control cohorts were therefore combined for each cell type for Fig3 in the main manuscript.

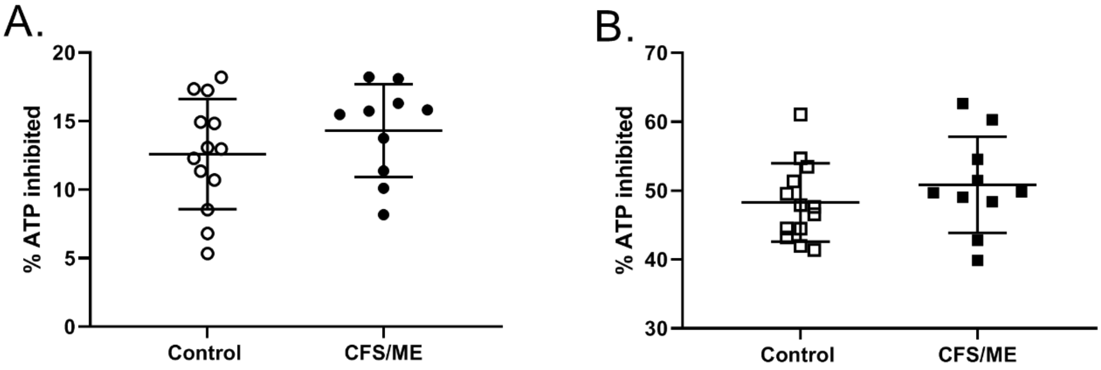

Figure S3. Results from healthy control and CFS/ME neutrophils and PBMCs on the percentage of ATP inhibited when sodium azide was added. (A) N*eutrophils (p=0.290).* ***(B)*** *PBMCs (p=0.343). Groups were compared using Student's t-tests. Significance level p≤0.01.*
